# A small molecule CFTR potentiator restores ATP‐dependent channel gating to the cystic fibrosis mutant G551D‐CFTR

**DOI:** 10.1111/bph.15709

**Published:** 2022-01-21

**Authors:** Jia Liu, Allison P. Berg, Yiting Wang, Walailak Jantarajit, Katy J. Sutcliffe, Edward B. Stevens, Lishuang Cao, Marko J. Pregel, David N. Sheppard

**Affiliations:** ^1^ Neuroscience and Pain Research Unit Pfizer Inc. Cambridge UK; ^2^ School of Physiology, Pharmacology and Neuroscience University of Bristol Bristol UK; ^3^ Rare Disease Research Unit Pfizer Inc. Cambridge MA USA; ^4^ Center of Calcium and Bone Research and Department of Physiology, Faculty of Science Mahidol University Bangkok Thailand

**Keywords:** cystic fibrosis transmembrane conductance regulator (CFTR), CFTR potentiation, chloride ion channel, cystic fibrosis, F508del‐CFTR, G551D‐CFTR, ivacaftor (VX‐770)

## Abstract

**Background and Purpose:**

Cystic fibrosis transmembrane conductance regulator (CFTR) potentiators are small molecules developed to treat the genetic disease cystic fibrosis (CF). They interact directly with CFTR Cl^−^ channels at the plasma membrane to enhance channel gating. Here, we investigate the action of a new CFTR potentiator, CP‐628006 with a distinct chemical structure.

**Experimental Approach:**

Using electrophysiological assays with CFTR‐expressing heterologous cells and CF patient‐derived human bronchial epithelial (hBE) cells, we compared the effects of CP‐628006 with the marketed CFTR potentiator ivacaftor.

**Key Results:**

CP‐628006 efficaciously potentiated CFTR function in epithelia from cultured hBE cells. Its effects on the predominant CFTR variant F508del‐CFTR were larger than those with the gating variant G551D‐CFTR. In excised inside‐out membrane patches, CP‐628006 potentiated wild‐type, F508del‐CFTR, and G551D‐CFTR by increasing the frequency and duration of channel openings. CP‐628006 increased the affinity and efficacy of F508del‐CFTR gating by ATP. In these respects, CP‐628006 behaved like ivacaftor. CP‐628006 also demonstrated notable differences with ivacaftor. Its potency and efficacy were lower than those of ivacaftor. CP‐628006 conferred ATP‐dependent gating on G551D‐CFTR, whereas the action of ivacaftor was ATP‐independent. For G551D‐CFTR, but not F508del‐CFTR, the action of CP‐628006 plus ivacaftor was greater than ivacaftor alone. CP‐628006 delayed, but did not prevent, the deactivation of F508del‐CFTR at the plasma membrane, whereas ivacaftor accentuated F508del‐CFTR deactivation.

**Conclusions and Implications:**

CP‐628006 has distinct effects compared to ivacaftor, suggesting a different mechanism of CFTR potentiation. The emergence of CFTR potentiators with diverse modes of action makes therapy with combinations of potentiators a possibility.

**Abbreviations**
FRT cells: Fischer rat thyroid cells
i: single‐channel current amplitude
IBI: interburst interval
I_Cl_^apical^
: apical Cl^−^ current
I_sc_
: short‐circuit current
MBD: mean burst duration
NBD: nucleotide‐binding domain
P_o_
: open probability
P_o(app)_
: apparent open probability
R_t_
: transepithelial resistance
YFP: yellow fluorescent protein

**What is already known**

- The marketed cystic fibrosis drug ivacaftor restores function to mutant CFTR by potentiating channel gating.

**What does this study add**

- CP‐628006, a new CFTR potentiator, efficaciously enhances CFTR function in cystic fibrosis patient‐derived airway cells.
- The action of CP‐628006 on mutant CFTR gating is distinct from that of ivacaftor.

**What is the clinical significance**

- Greater clinical benefit in cystic fibrosis might be achieved with combinations of CFTR potentiators.

## 1 INTRODUCTION

Treatment of the life‐limiting genetic disease cystic fibrosis (CF) has been transformed by the orally bioavailable drug (ivacaftor VX‐770; Vertex Pharmaceuticals) (Ramsey et al., 2011; Ratjen et al., 2015; Van Goor et al., 2009). Ivacaftor was the first drug to target the root cause of cystic fibrosis, pathogenic variants in the cystic fibrosis transmembrane conductance regulator (CFTR) (Riordan et al., 1989). CFTR is an ATP‐binding cassette transporter that functions as an ATP‐gated anion channel activated by protein kinase A (PKA)‐dependent phosphorylation (Hwang et al., 2018). Widely expressed in epithelial tissues, CFTR plays a pivotal role in fluid and electrolyte movements, regulating the quantity and composition of epithelial secretions (Saint‐Criq & Gray, 2017).

Ivacaftor is a CFTR potentiator, an agent that increases the activity of mutant Cl^−^ channels present at the plasma membrane (Jih et al., 2017). It does not open silent channels, but once CFTR is phosphorylated by PKA, ivacaftor increases the frequency and duration of channel openings by modifying ATP‐dependent and ATP‐independent channel gating (Eckford et al., 2012; Jih & Hwang, 2013). Ivacaftor treatment of individuals with cystic fibrosis and CFTR gating variants (e.g., G551D; Bompadre et al., 2007; Cai et al., 2006; Gregory et al., 1991; Xu et al., 2014) achieves sustained, long‐term clinical benefit, including a slower decline in lung function and improved nutrition (Volkova et al., 2020). In combination with CFTR correctors, which traffic F508del‐CFTR to the plasma membrane, ivacaftor also has clinical benefit for cystic fibrosis patients with the predominant F508del variant (e.g., Heijerman et al., 2019; Middleton et al., 2019).

**FIGURE 1:**
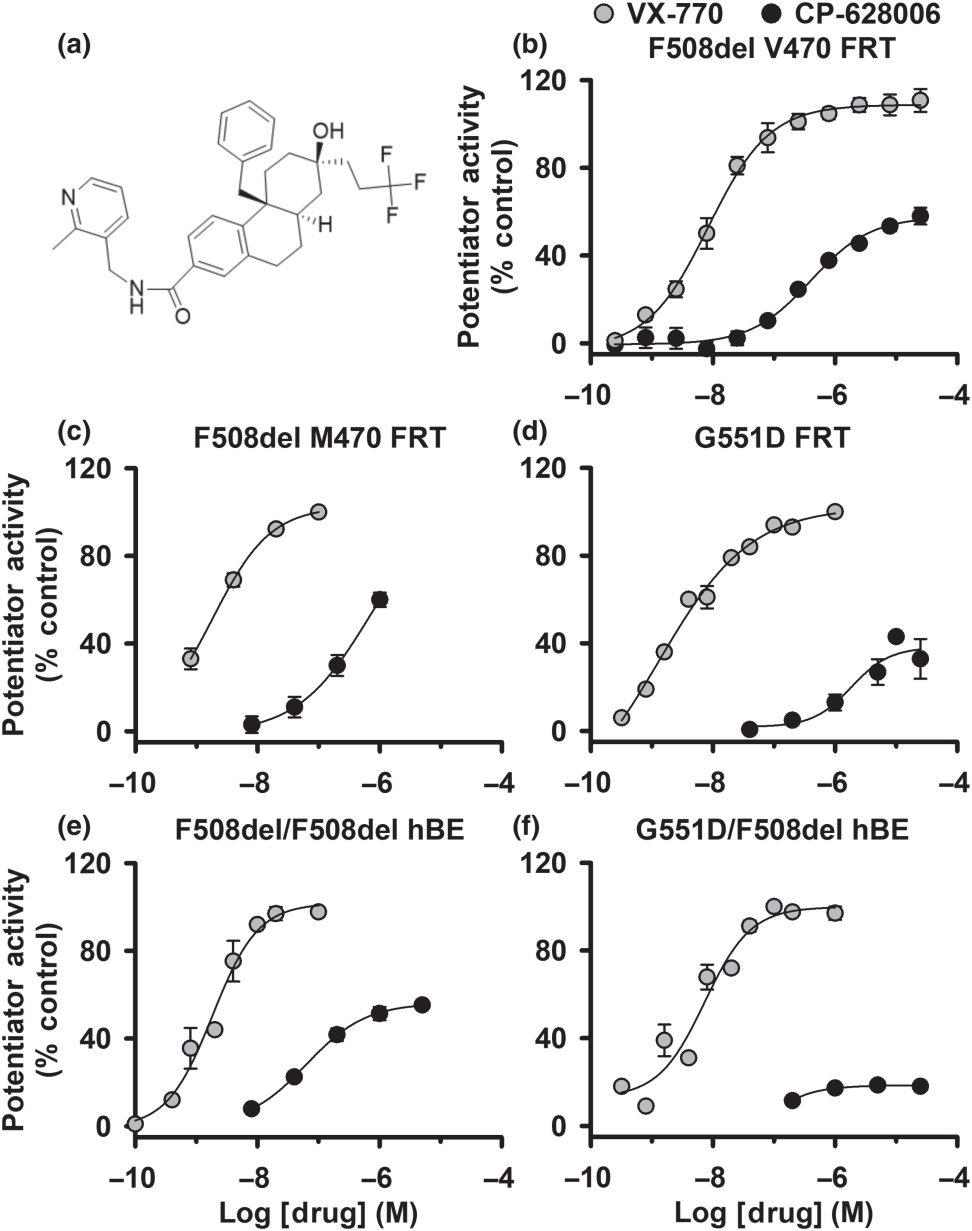
Exploratory data. CP‐628006 potentiates F508del‐ and G551D‐CFTR in FRT cells heterologously expressing CFTR and in human bronchial epithelial cells expressing native CFTR. (a) Chemical structure of CP‐628006. (b–f) Concentration–response relationships for F508del‐ and G551D‐CFTR potentiation by CP‐628006 and ivacaftor (VX‐770) determined by the fit of least squares functions using a 4‐parameter (variable slope) model with the minimum response set to zero. In (b), potentiation by CP‐628006 and ivacaftor are expressed relative to the responses achieved by DMSO (0.25% v·v^−1^) (0%) and genistein (50 μM) (100%). In (c–f), potentiation by CP‐628006 is expressed relative to that of ivacaftor with the ivacaftor concentration achieving maximal response designated as 100% (in μM: c, 0.1; d, 1; e, 0.02; and f, 0.2). Data are means ± SEM, except where indicated and the continuous lines are four parameter logistical fits to mean data. (b) CFTR‐mediated anion transport by FRT cells co‐expressing F508del‐V470‐CFTR and YFP determined by I^−^‐induced quenching of YFP fluorescence (CP‐628006, *n* = 6; ivacaftor, *n* = 6). (c, d) CFTR‐mediated apical Cl^−^ currents (I_Cl_^apical^) in FRT epithelia heterologously expressing F508del‐M470‐ and G551D‐CFTR (F508del‐CFTR: CP‐628006, *n* = 1; ivacaftor, *n* = 3; G551D‐CFTR: CP‐628006, *n* = 4; ivacaftor, *n* = 4). (e, f) CFTR‐mediated short‐circuit current (I_sc_) from epithelia of human bronchial epithelial cells with genotypes F508del/F508del and F508del/G551D (F508del/F508del: CP‐628006, *n* = 3; ivacaftor, *n* = 3; F508del/G551D: CP‐628006, *n* = 2; ivacaftor, *n* = 9). In (c)–(f), CFTR_inh_‐172 (20 μM) was added after test potentiators to inhibit CFTR‐mediated I_Cl_^apical^ and I_sc_

Here, we investigated the action of CP‐628006 (Murry & White, 2002), a small molecule with a chemical structure distinct from that of ivacaftor and other previously reported CFTR potentiators (Figure 1a). It was discovered in a high‐throughput screen of a compound library to identify new CFTR potentiators. To characterise CP‐628006, we used mammalian cells heterologously expressing CFTR and patient‐derived primary cultures of human bronchial epithelial cells expressing native CFTR. With Ussing chambers, automated planar patch‐clamp and single‐channel recording, we compared CFTR potentiation by CP‐628006 with ivacaftor at the tissue, cellular and molecular levels. Our results demonstrate that CP‐628006 is an efficacious CFTR potentiator with distinct effects compared to those of ivacaftor, suggesting a different mechanism of action.

## 2 METHODS

### 2.1 Cells and cell culture

Fischer rat thyroid (FRT) epithelial cells (RRID:CVCL_A6IE) expressing CFTR variants were used for functional studies with halide flux and Ussing chamber techniques. FRT cells stably expressing the F508del variant with the V470 polymorphism and the halide sensor yellow fluorescent protein (YFP)‐H148Q/I152L (F508del‐CFTR‐YFP‐FRT cells) (Galietta et al., 2001) were obtained from LJV Galietta (Telethon Institute for Genetics and Medicine, Pozzuoli, Italy). They were cultured in Coon's modified Ham's F‐12 medium supplemented with 10% FBS, 2‐mM GlutaMAX™, 0.27% NaHCO_3_, 100 U·ml^−1^ penicillin and 100 μg·ml^−1^ streptomycin and 0.8 mg·ml^−1^ zeocin at 37°C in a humidified atmosphere with 5% CO_2_. For halide flux studies, F508del‐CFTR‐YFP‐FRT cells were seeded in 384‐well plates at a density of 25,000 cells per well in 50 μl per well of media. After 18–24 h culture at 37°C, the temperature was reduced to 27°C for 16–24 h to promote the plasma membrane expression of F508del‐CFTR (Denning et al., 1992).

Ussing chamber studies to determine the concentration–response relationships of CFTR potentiators (Figure 1c–f) were performed at Charles River Laboratories (ChanTest) (Cleveland, OH, USA). For these studies, FRT cells were stably transfected with CFTR cDNAs with the F508del and G551D variants and the common polymorphism M470 (synthesised by System Biosciences, Palo Alto, CA, USA) using the Flp‐In™ system (Thermo Fisher Scientific, Waltham, MA, USA). The F508del‐CFTR cDNA contained the following differences from the canonical CFTR cDNA sequence (NCBI Accession Number NM_000492): del CTT (1521–1523) to delete F508; synonymous changes T > C (798), A > G (801), T > C (804) to remove a cryptic bacterial promoter; synonymous change T > C (1095); G > A (1408) to give M470 and was amplified using the primers 5′ gttgctagcggtaccatgcagaggtcgcctctgga 3′ and 5′ gttgcggccgcctaaagccttgtatcttgcacctc 3′. The G551D‐CFTR cDNA contained the following differences from the canonical CFTR cDNA sequence (NCBI Accession Number NM_000492): G > A (1652) to replace G551 with D551; synonymous changes T > C (798), A > G (801), T > C (804) to remove a cryptic bacterial promoter; synonymous change A > C (516); synonymous change T > C (1095); G > A (1408) to give M470 and was amplified using the primers 5′ gttgctagcggtaccatgcagaggtcgcctctgga 3′ and 5′ gttgcggccgcctaaagccttgtatcttgcacctc 3′. Using Kpn1 and Not1 restriction sites, the F508del‐ and G551D‐CFTR cDNAs were inserted into pcDNA5/FRT (Flp‐In™) (RRID:Addgene_31984). An FRT cell line stably expressing a flippase (Flp)‐recombinase recognition target integration site (generated by Evotec AG, Hamburg, Germany, using isogenic FRT cells from LJV Galietta) was transfected with the F508del‐ and G551D‐CFTR plasmids and the Flp recombinase plasmid *pOG44* (RRID:Addgene_129419) following the manufacturer's instructions. To identify FRT cells expressing F508del‐ and G551D‐CFTR, cells were grown under hygromycin selection and individual clones picked, expanded, and verified for CFTR expression. For Ussing chamber studies to determine the plasma membrane stability of F508del‐CFTR, FRT cells expressing F508del‐CFTR from LJV Galietta were cultured and used as described previously (Meng et al., 2017).

For functional studies of native CFTR, we used primary cultures of human bronchial epithelial cells of genotypes F508del/F508del and F508del/G551D. Passage 1 (P1) human bronchial epithelial cells from SH Randell (University of North Carolina [UNC] Cystic Fibrosis Center Tissue Procurement and Cell Culture Core) were differentiated at an air–liquid interface as described by Neuberger et al., (2011) with some modifications. The research conducted on human cells at UNC has been verified as compliant with Pfizer policies, including institutional review board or institutional ethics committee approval. Cells were thawed and expanded in BEGM™ Bronchial Epithelial Cell Growth Medium (cat. no. CC‐3170, Lonza, USA) supplemented with retinoic acid (10 nM) and banked at P2. For functional assays, P2 cells were expanded to P4, and 5 × 10^5^ P4 human bronchial epithelial cells were seeded in growth medium on Snapwell filter inserts (cat. no. 3801, Corning, NY, USA). Cells were switched to differentiation medium the following day and cultured submerged for 3 days before the medium was removed from the apical membrane to establish an air–liquid interface. Thereafter, the basolateral medium was changed every 2–3 days and mucus washed from the apical membrane every 2–3 weeks and the day prior to use starting 21 days after initiating air–liquid interface culture.

For CFTR studies using automated electrophysiology and single‐channel recording, we used baby hamster kidney (BHK) cells (RRID:CVCL_1915) and human embryonic kidney (HEK) 293 cells (RRID:CVCL_0045) stably expressing wild‐type human CFTR and the CFTR variants F508del‐ and G551D‐CFTR (Farinha et al., 2002; Xu et al., 2014). BHK cells from MD Amaral (University of Lisboa) were cultured and used as described previously (Schmidt et al., 2008) with the exception that the plasma membrane expression of F508del‐CFTR was rescued by either low temperature incubation (27°C for 72–96 h) or treatment with the CFTR corrector lumacaftor (3 μM for 24 h at 37°C) (Van Goor et al., 2011). HEK cells expressing CFTR variants were generated using the Flp‐In™ system as described above for F508del‐ and G551D‐CFTR‐expressing FRT cells. They were cultured in Dulbecco's modified Eagle's medium (glucose, 1.5 g·L^−1^; sodium pyruvate, 110 mg·L^−1^) supplemented with 10% FBS (PAA Laboratories), GlutaMAX™ and hygromycin‐B (150 μg·ml^−1^; Invitrogen™, Thermo Fisher Scientific) at 37°C in a humidified atmosphere with 5% CO_2_. Prior to study, F508del‐CFTR expressed in HEK cells was trafficked to the plasma membrane by low temperature incubation (27°C for 24–72 h). The single‐channel behaviour of human CFTR in excised membrane patches from different mammalian cell lines heterologously expressing CFTR is equivalent (wild‐type CFTR, Chen et al., 2009; F508del‐CFTR, Bose et al., 2019; and G551D‐CFTR, J Liu, Y Wang, Z Cai, and DN Sheppard, unpublished observations).

### 2.2 Fluorescence‐based halide flux assay

CFTR‐mediated anion transport was quantified by measuring I^−^‐induced quenching of YFP fluorescence using a microtitre plate reader following the method of Galietta et al. (2001). Cells were washed twice with phosphate‐buffered saline (PBS) (composition [mM]: 137 NaCl, 2.7 KCl, 8.1 Na_2_HPO_4_, 1.5 KH_2_PO_4_, 1 CaCl_2_, and 0.5 MgCl_2_, pH 7.40). Then, cells were incubated with forskolin (20 μM) and test small molecules (serial dilutions, maximum concentration 30 μM) or an equivalent volume of the vehicle DMSO in 30 μl of PBS for 30 min at 23°C. Using a fluorescence microtitre plate reader (FLIPR384; Molecular Devices, San Jose, CA, USA) equipped with 500‐nm excitation and 535‐nm emission filters, an initial fluorescence reading was recorded before addition of PBS‐iodide solution (composition [mM]: 137 NaI, 2.7 KCl, 8.1 Na_2_HPO_4_, 1.5 KH_2_PO_4_, 1 CaCl_2_, and 0.5 MgCl_2_, pH 7.40) into each well and 21 s later a second fluorescence reading was acquired. To quantify CFTR‐mediated anion transport, the second fluorescence value (*F*) was expressed relative to the fluorescence value immediately before PBS‐iodide addition (*F*_0_) and the resulting value then expressed relative to the responses of DMSO and genistein (50 μM) designated as the 0% and 100% responses, respectively.

### 2.3 Ussing chamber studies

#### 2.3.1 FRT cells heterologously expressing CFTR

To grow FRT cells as polarised epithelia, 2 × 10^5^ FRT cells were seeded on Snapwell filter inserts and cultured for 7–14 days before use; media were changed every 2–3 days. We studied FRT epithelia with transepithelial resistance (R_t_) of >1000 Ω cm^2^ and rescued the plasma membrane expression of F508del‐CFTR by incubating F508del‐CFTR‐expressing FRT epithelia with lumacaftor (5 μM) for 18–24 h at 37°C prior to use.

FRT epithelia were mounted in Ussing chambers (Physiologic Instruments Inc., San Diego, CA, USA). The solution bathing the basolateral membrane contained (mM): 137 NaCl, 4 KCl, 1.8 CaCl_2_, 1 MgCl_2_, 10 HEPES, and 10 glucose adjusted to pH 7.35 with NaOH. The solution bathing the apical membrane was identical to that of the basolateral solution with the exception that (mM): 68.5 Na gluconate + 68.5 NaCl replaced 137‐mM NaCl to create a Cl^−^ concentration gradient (basolateral [Cl^−^], 146.6 mM; apical [Cl^−^], 78.1 mM). All solutions were maintained at 27°C to sustain the apical membrane expression of F508del‐CFTR and bubbled continuously with air under low pressure to circulate buffers and mix test compounds.

To test the effects of CP‐628006 on CFTR Cl^−^ channels in the apical membrane of FRT epithelia, we permeabilized the basolateral membrane with amphotericin B (100 μM). After cancelling voltage offsets, we clamped transepithelial voltage at 0 mV and measured apical Cl^−^ current (I_Cl_^apical^) continuously using a Physiologic Instruments VCC MC6/8 multichannel voltage/current clamp (Li et al., 2004). After recording basal I_Cl_^apical^ for ~15 min, we added forskolin (10 μM, apical and basolateral solutions), test CFTR potentiators (four sequential additions to the apical solution), and the CFTR inhibitor CFTR_inh_‐172 (20 μM, apical and basolateral solutions) sequentially and cumulatively at 20‐ to 25‐min intervals. The resistance of the filter and solutions, in the absence of cells, was subtracted from all measurements. Under the experimental conditions used, current flow from basolateral to apical solutions corresponds to Cl^−^ movement through open CFTR Cl^−^ channels and is shown as an upward deflection.

To acquire and analyse I_Cl_^apical^, we used LabScribe v2 software (iworx, Dover, NH, USA). The CP‐628006 responses were scaled relative to those of DMSO (0%) and the maximal ivacaftor response (100%). To determine half‐maximal concentrations (EC_50_) values for CFTR potentiation, we constructed concentration–response relationships. With GraphPad Prism (La Jolla, CA, USA) (RRID:SCR_002798), I_Cl_^apical^ responses were plotted against the logarithm of the compound concentration and EC_50_ values derived by fitting least squares functions using a 4‐parameter (variable slope) model with the minimum response constrained to zero.

#### 2.3.2 Cystic fibrosis human bronchial epithelia

After >30 days of air–liquid interface culture on Snapwell filter inserts, human bronchial epithelial cells formed well differentiated, electrically tight epithelia (R_t_ > 200 Ω cm^2^). Prior to study, F508del/F508del and F508del/G551D human bronchial epithelial cell cultures were incubated at 37°C and 5% CO_2_.

Epithelia were mounted in Ussing chambers and data acquired and analysed as described above for Ussing chamber studies of FRT cells heterologously expressing CFTR with the following exceptions. Both the apical and basolateral membranes were bathed in solutions containing (mM): 137 NaCl, 4 KCl, 1.8 CaCl_2_, 1 MgCl_2_, 10 Na HEPES, and 10 glucose (pH 7.4 with NaOH). Epithelia of F508del/G551D human bronchial epithelial cells were studied at 35°C, whereas those of F508del/F508del human bronchial epithelial cells were assayed at 27°C to maintain the apical membrane expression of F508del‐CFTR. After cancelling voltage offsets, transepithelial voltage was clamped at 0 mV and short‐circuit current (I_sc_) recorded continuously. The protocol to record CFTR‐mediated I_sc_ was the same as for I_Cl_^apical^ measurements except that amiloride (30 μM, apical solution) was added to inhibit the epithelial Na^+^ channel before forskolin (10 μM, apical and basolateral solutions) addition.

#### 2.3.3 F508del‐CFTR plasma membrane stability studies

To evaluate the effects of CP‐628006 on the plasma membrane stability of F508del‐CFTR, we used a modification of the method described by Meng et al. (2017). After 48‐h incubation at 27°C to rescue the plasma membrane expression of F508del‐CFTR, FRT epithelia were treated with cycloheximide (50 μg·ml^−1^) and DMSO or test small molecules, 15 min prior to *t* = 0 h. At *t* = 0, 2, 4, and 6 h after these treatments, F508del‐CFTR expressing FRT epithelia were mounted in Ussing chambers (Warner Instrument Corp., Dual Channel Chamber; Harvard Apparatus Ltd., Edenbridge, UK) and I_sc_ responses recorded.

CFTR‐mediated I_sc_ was recorded using a large Cl^−^ concentration gradient without permeabilizing the basolateral membrane. The solution bathing the basolateral membrane contained (mM): 140 NaCl, 5 KCl, 0.36 K_2_HPO_4_, 0.44 KH_2_PO_4_, 1.3 CaCl_2_, 0.5 MgCl_2_, 10 HEPES, and 4.2 NaHCO_3_, adjusted to pH 7.2 with Tris ([Cl^−^], 149 mM). The solution bathing the apical membrane was identical to that of the basolateral solution with the exception that (mM): 133.3 Na gluconate + 2.5 NaCl and 5 K gluconate replaced 140 NaCl and 5 KCl, respectively, ([Cl^−^], 14.8 mM). To compensate for the calcium buffering capacity of gluconate, we used 5.7‐mM Ca^2+^ in the apical solution. All solutions were maintained at 37°C and bubbled continuously with 5% CO_2_.

After cancelling voltage offsets, we clamped transepithelial voltage (referenced to the basolateral solution) at 0 mV and recorded I_sc_ continuously using an epithelial voltage‐clamp amplifier (Warner Instrument Corp., model EC‐825; Harvard Apparatus Ltd.), digitising data as described previously (Li et al., 2004). The resistance of the filter and solutions, in the absence of cells, was subtracted from all measurements. For illustration purposes, I_sc_ time courses are displayed as ΔI_sc_ with the I_sc_ value immediately preceding forskolin addition designated as 0 μA/cm^2^; file sizes were compressed by 100‐fold data reduction.

### 2.4 Single‐channel recording

CFTR Cl^−^ channels were recorded in excised inside‐out membrane patches using an Axopatch 200B patch‐clamp amplifier and pCLAMP software (version 10.3) (RRID:SCR_011323) (both from Molecular Devices, San Jose, CA, USA) (Sheppard & Robinson, 1997). The pipette (extracellular) solution contained (mM): 140 *N*‐methyl‐d‐glucamine (NMDG), 140 aspartic acid, 5 CaCl_2_, 2 MgSO_4_, and 10 *N*‐tris[hydroxymethyl]methyl‐2‐aminoethanesulphonic acid (TES), adjusted to pH 7.3 with Tris ([Cl^−^], 10 mM). The bath (intracellular) solution contained (mM): 140 NMDG, 3 MgCl_2_, 1 CsEGTA, and 10 TES, adjusted to pH 7.3 with HCl ([Cl^−^], 147 mM; free [Ca^2+^], <10^−8^ M) at room temperature (~23°C) unless otherwise indicated.

Within 2 min after membrane patch excision, we added the catalytic subunit of protein kinase A (PKA; 75 nM) and ATP (1 mM) to the intracellular solution to activate CFTR Cl^−^ channels. To minimise channel rundown, we added PKA and millimolar concentrations of ATP to all intracellular solutions and clamped voltage at −50 mV. CP‐628006 and ivacaftor were studied by addition to the intracellular solution in the continuous presence of ATP (1 mM [F508del‐ and G551D‐CFTR] or 0.3 mM [wild‐type]) and PKA (75 nM). We reduced the ATP concentration when testing CFTR potentiators on wild‐type CFTR to observe their effects more clearly. Because of the difficulty of washing ivacaftor from the recording chamber (Wang et al., 2014), test interventions were not bracketed by control periods. Instead, specific interventions with CFTR potentiators were compared with the pre‐intervention control period made with the same concentration of ATP and PKA, but without test compounds. To investigate the ATP‐dependence of CFTR potentiators, CP‐628006 and ivacaftor were studied in different excised membrane patches from those used to acquire control data and at least three ATP concentrations were tested in each experiment.

To investigate the effects of CFTR potentiators on the plasma membrane stability of F508del‐CFTR, we monitored its thermal stability in excised inside‐out membrane patches at 37°C (Wang et al., 2014). Membrane patches were excised and F508del‐CFTR Cl^−^ channels activated at 27°C. Once channels were fully activated and potentiated, the temperature of the intracellular solution was increased to 37°C, which took 2–3 min. To quantify data, we calculated open probability (P_o_) and normalised P_o_ values in 30‐s intervals over a 9‐min period (Wang et al., 2014).

**FIGURE 2:**
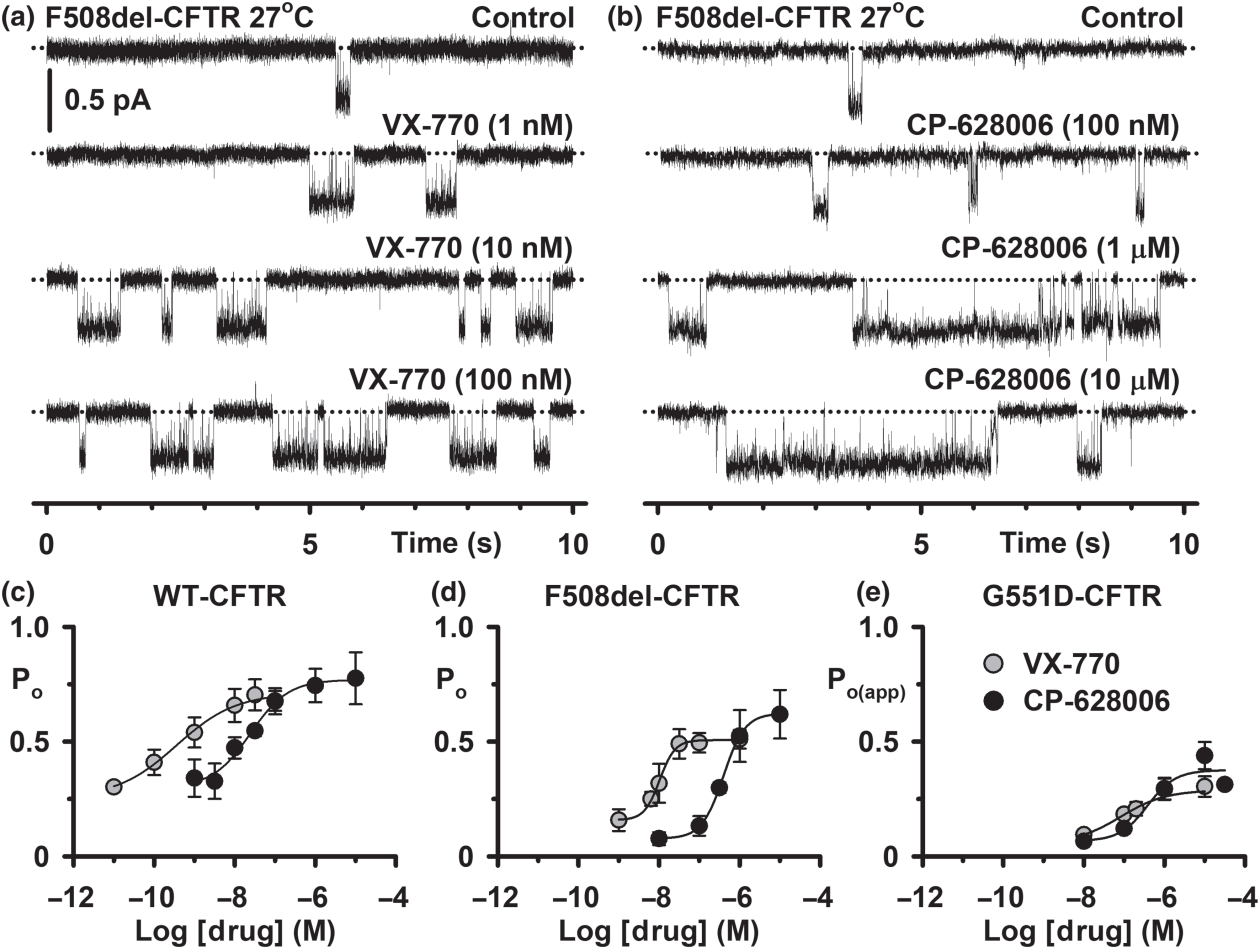
CP‐628006 potentiates the single‐channel activity of wild‐type, F508del‐ and G551D‐CFTR. (a, b) Representative single‐channel recordings of low temperature‐rescued F508del‐CFTR in excised inside‐out membrane patches from HEK293 cells show the effects of acute addition of the indicated concentrations of ivacaftor (VX‐770) and CP‐628006 to the intracellular solution. ATP (1 mM) and PKA (75 nM) were continuously present in the intracellular solution. Dotted lines indicate the closed channel state and downward deflections correspond to channel openings. Unless otherwise indicated in this and other figures with single‐channel data, membrane patches were voltage‐clamped at −50 mV, a large Cl^−^ concentration gradient was imposed across the membrane ([Cl^−^]_int_, 147 mM; [Cl^−^]_ext_, 10 mM) and temperature was ~23°C. (c–e) Concentration–response relationships for CFTR potentiation by CP‐628006 and ivacaftor. Data are P_o_ and P_o(app)_ values for wild‐type, low temperature‐rescued F508del‐ and G551D‐CFTR determined from prolonged recordings acquired using the conditions described in (a) and (b) (wild‐type, ≥5 min; F508del‐ and G551D‐CFTR, ≥10 min). G551D‐CFTR data are presented as P_o(app)_ because the number of channels in membrane patches is unknown. Data are means ± SEM (wild‐type CFTR: CP‐628006, *n* = 3–4; ivacaftor, *n* = 4–5; 27°C‐rescued F508del‐CFTR: CP‐628006, *n* = 5–12; ivacaftor, *n* = 4–7; G551D‐CFTR: CP‐628006, *n* = 4–9; ivacaftor, *n* = 4–13). The continuous lines are the fit of sigmoidal concentration‐response functions to mean data. To determine EC_50_ and maximal effect values (Table 1), potentiation by CP‐628006 is expressed relative to that of ivacaftor with the ivacaftor concentration achieving maximal effect (1 μM) designated 100%

In this study, we used membrane patches containing ≤5 active channels (wild‐type CFTR, number of active channels [*n* ≤ 3; F508del‐CFTR, *n* ≤ 4; G551D‐CFTR, *n* ≤ 5). To determine channel number, we used the maximum number of simultaneous channel openings observed during an experiment (Cai et al., 2006). To minimise errors, we used experimental conditions that robustly potentiate channel activity and verified that recordings were of sufficient length to ascertain the correct number of channels (Venglarik et al., 1994). For G551D‐CFTR, single‐channel recordings lasting around 10 min per intervention were used for the data presented in Figures 2 and 6. For the G551D‐CFTR data presented in Figure 4, the duration of recordings and number of events analysed were as follows: control: duration, 454 ± 77 s; no. events, 190 ± 89; CP‐628006: duration, 718 ± 165 s; no. events, 4149 ± 1523; ivacaftor: duration, 432 ± 78 s; no. events, 12,710 ± 4829; CP‐628006 + ivacaftor: duration, 927 ± 335 s; no. events, 17,753 ± 4324 (*n* = 6, except CP‐628006, where *n* = 5). Despite our precautions, we cannot exclude the possibility of unobserved G551D‐CFTR Cl^−^ channels in excised membrane patches causing P_o_ values for G551D‐CFTR to be overestimated. Therefore, P_o_ values for G551D‐CFTR are expressed as apparent P_o_ (P_o(app)_) values.

Single‐channel currents were directly acquired to a computer hard disc after filtering at a corner frequency (*f*_c_) of 500 Hz with an eight‐pole Bessel filter (model LHBF‐48×; npi electronic GmbH, Tamm, Germany) and digitising at a sampling rate of 5 kHz using a Digidata 1440A interface (Molecular Devices) and pCLAMP software. To measure single‐channel current amplitude (i), Gaussian distributions were fit to current amplitude histograms. For P_o_ and burst analyses, lists of open‐ and closed‐times were created using a half‐amplitude crossing criterion for event detection and dwell‐time histograms constructed as previously described (Sheppard & Robinson, 1997); transitions < 1 ms were excluded from the analysis (eight‐pole Bessel filter rise time [*T*_10–90_] ~ 0.73 ms at *f*_c_ = 500 Hz). Histograms were fitted with one or more component exponential functions using the maximum likelihood method. For burst analyses, we used a *t*_c_ (the time that separates interburst closures from intraburst closures) determined from closed time histograms (low temperature‐rescued F508del‐CFTR: control, *t*_c_ = 25.4 ± 1.7 ms [*n* = 6]; CP‐628006, *t*_c_ = 20.2 ± 3.9 ms [*n* = 5]; ivacaftor, *t*_c_ = 29.1 ± 7.5 ms [*n* = 4]) (Cai et al., 2006). The mean interburst interval (T_IBI_) was calculated using the equation (Cai et al., 2006):

(1) $$P_{o}=T_{b}/\left(T_{\mathrm{MBD}}+T_{\mathrm{IBI}}\right),$$

where *T*_b_ = (mean burst duration) × (open probability within a burst). Mean burst duration (*T*_MBD_) and open probability within a burst (P_o[burst]_) were determined directly from experimental data using pCLAMP software. Only membrane patches that contained a single active channel were used for burst analysis. For illustration purposes, single‐channel recordings were filtered at 500 Hz and digitised at 5 kHz before file size compression by five‐fold data reduction.

### 2.5 Materials

The CFTR potentiator CP‐628006 was synthesised by Pfizer (Murry & White, 2002). Ivacaftor and lumacaftor were synthesised by Pfizer or purchased from either Selleck Chemicals (Houston, TX, USA) or Cayman Chemicals (Ann Arbor, MI, USA). PKA purified from bovine heart was purchased from Calbiochem (Merck Chemicals Ltd., Nottingham, UK). All other chemicals were of reagent grade and supplied by MilliporeSigma (now Merck Life Science UK Ltd.) (St Louis, MO, USA).

ATP was dissolved in intracellular solution, forskolin in DMSO (automated electrophysiology) or methanol (Ussing chamber stability studies), while all other reagents were dissolved in DMSO. Stock solutions were stored at −20°C except for that of ATP, which was prepared freshly before each experiment. Immediately before use, stock solutions were diluted to final concentrations and, where necessary in patch‐clamp experiments, the pH of the intracellular solution was readjusted to pH 7.3 to avoid pH‐dependent changes in CFTR function (Chen et al., 2009). Precautions against light‐sensitive reactions were observed when using CFTR modulators. DMSO was without effect on CFTR activity (Schmidt et al., 2008; Sheppard & Robinson, 1997). On completion of experiments, the recording chamber was thoroughly cleaned before re‐use as described previously (Meng et al., 2017; Wang et al., 2014).

### 2.6 Statistics

**FIGURE 3:**
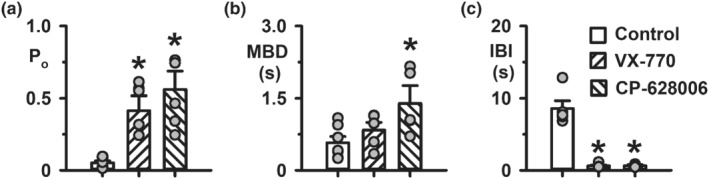
CP‐628006 increases the frequency and duration of low temperature‐rescued F508del‐CFTR Cl^−^ channel openings. (a–c) Open probability (P_o_), mean burst duration (MBD), and interburst interval (IBI) of low temperature‐rescued F508del‐CFTR in the absence and presence of CP‐628006 (10 μM) and ivacaftor (VX‐770; 1 μM) added acutely to the intracellular solution bathing excised inside‐out membrane patches. Data are from membrane patches containing a single active F508del‐CFTR Cl^−^ channel acquired using ATP (1 mM) and PKA (75 nM). Because some control recordings were unsuitable for burst analysis, control data from experiments with CP‐628006 and ivacaftor have been combined (CP‐628006 control, *n* = 2; ivacaftor control *n* = 5). Symbols represent individual values and columns are means ± SEM (control, *n* = 7; CP‐628006, *n* = 5; ivacaftor, *n* = 5); *P < 0.05 versus control; one‐way ANOVA with Dunnett's post hoc test. (a) Normality test (Shapiro–Wilk), *P* = 0.711 (passed); equal variance test (Brown‐Forsythe), *P* = 0.102 (passed); (b) normality test (Shapiro–Wilk), *P* = 0.602 (passed); equal variance test (Brown‐Forsythe), *P* = 0.160 (passed); (c) normality test (Shapiro–Wilk), *P* < 0.05 (failed)

**FIGURE 4:**
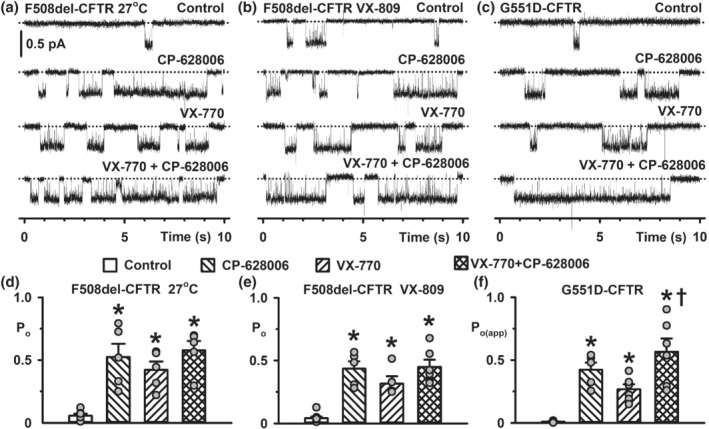
Potentiation of G551D‐CFTR Cl^−^ channels by a combination of CP‐628006 and ivacaftor is more efficacious than the maximal effect of either compound alone. (a–c) Representative single‐channel records of F508del‐CFTR rescued by low temperature incubation or lumacaftor (VX‐809) treatment and G551D‐CFTR in excised inside‐out membrane patches from HEK293 and BHK cells show the effects of CP‐628006 (10 μM) and ivacaftor (VX‐770) (F508del‐CFTR, 1 μM; G551D‐CFTR, 10 μM) added alone or together to the intracellular solution. ATP (1 mM) and PKA (75 nM) were continuously present in the intracellular solution. Dotted lines indicate the closed channel state and downward deflections correspond to channel openings. (d–f) Open probability (P_o_) and P_o(app)_ data for low temperature‐rescued F508del‐, lumacaftor‐rescued F508del‐, and G551D‐CFTR acquired using the conditions described in (a)–(c). Symbols represent individual values and columns are means ± SEM (27°C‐rescued F508del‐CFTR: control, *n* = 6; ivacaftor, *n* = 5; CP‐628006, *n* = 5; ivacaftor + CP‐628006, *n* = 5; lumacaftor‐rescued F508del‐CFTR: control, *n* = 9; ivacaftor, *n* = 5; CP‐628006, *n* = 5; ivacaftor + CP‐628006, *n* = 6; G551D‐CFTR: control, *n* = 6; ivacaftor, *n* = 6; CP‐628006, *n* = 5; ivacaftor + CP‐628006, *n* = 6); *P < 0.05 versus control; ^†^ *P* < 0.05 versus ivacaftor; one‐way ANOVA with Holm‐Sidak post hoc test. (d) Normality test (Shapiro–Wilk), *P* = 0.507 (passed); equal variance test (Brown‐Forsythe), *P* < 0.05 (failed); (e) normality test (Shapiro–Wilk), *P* = 0.287 (passed); equal variance test (Brown‐Forsythe), *P* = 0.076 (passed); (f) normality test (Shapiro–Wilk), *P* = 0.579 (passed); equal variance test (Brown‐Forsythe), *P* < 0.05 (failed). Other details as in Figure 2

**FIGURE 5:**
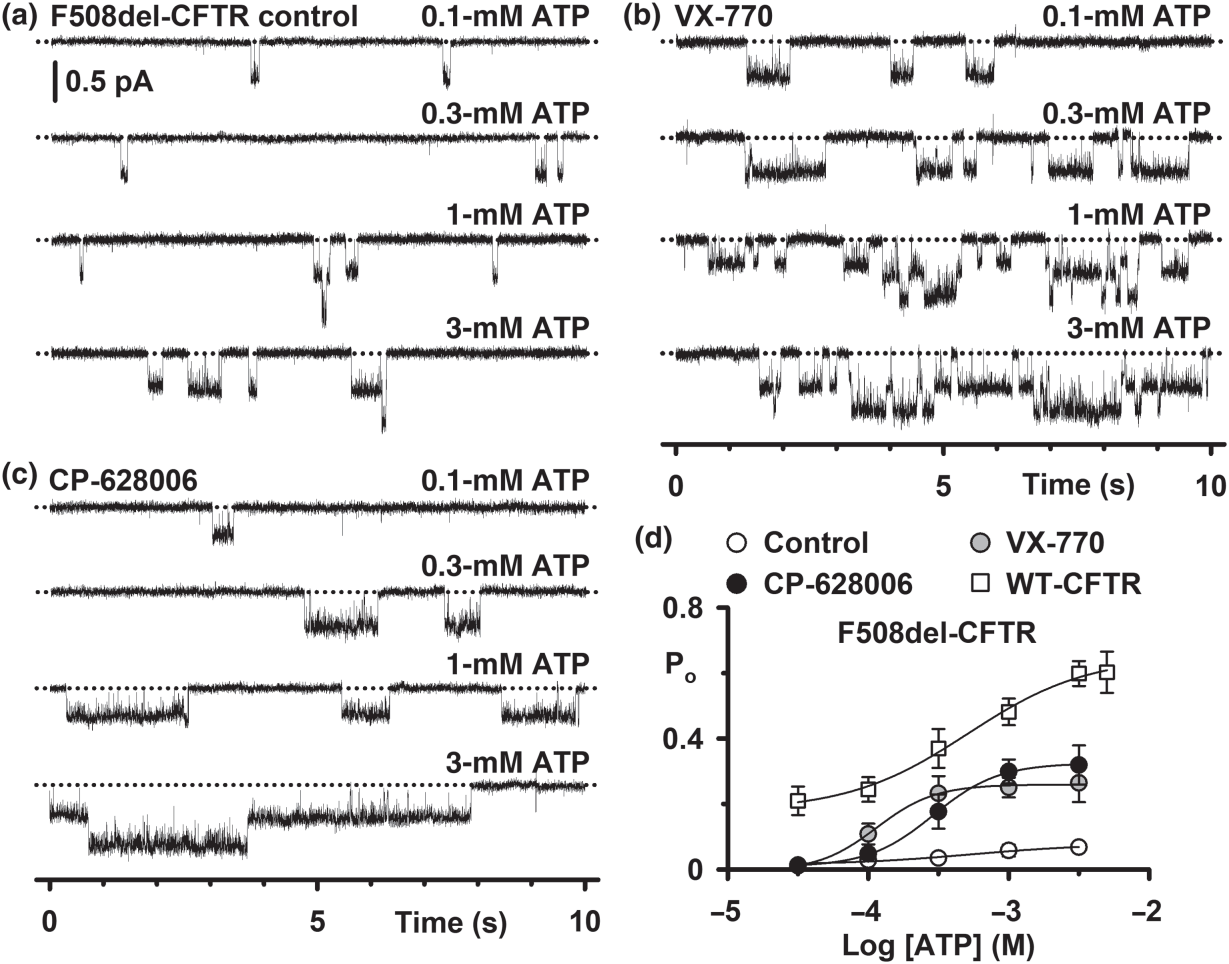
CP‐628006 and ivacaftor restore ATP‐dependent channel gating to low temperature‐rescued F508del‐CFTR. (a–c) Representative single‐channel recordings of low temperature‐rescued F508del‐CFTR acquired using different membrane patches excised from HEK293 cells show the effects of the indicated intracellular ATP concentrations on potentiation by ivacaftor (VX‐770; 7 nM) and CP‐628006 (300 nM). PKA (75 nM) was continuously present in the intracellular solution. Dotted lines indicate the closed channel state and downward deflections correspond to channel openings. (d) Relationship between intracellular ATP concentration and P_o_ for low temperature‐rescued F508del‐CFTR in the absence and presence of either CP‐628006 (300 nM) or ivacaftor (7 nM). For comparison, the relationship between intracellular ATP‐concentration and P_o_ of wild‐type CFTR phosphorylated with PKA (75 nM) is shown. Data are means ± SEM (wild‐type, *n* = 5–7; 27°C‐rescued F508del‐CFTR: control, *n* = 3; CP‐628006, *n* = 4–6; ivacaftor, *n* = 4–6). The continuous lines are the fit of sigmoidal concentration‐response functions to mean data. Other details as in Figure 2

**FIGURE 6:**
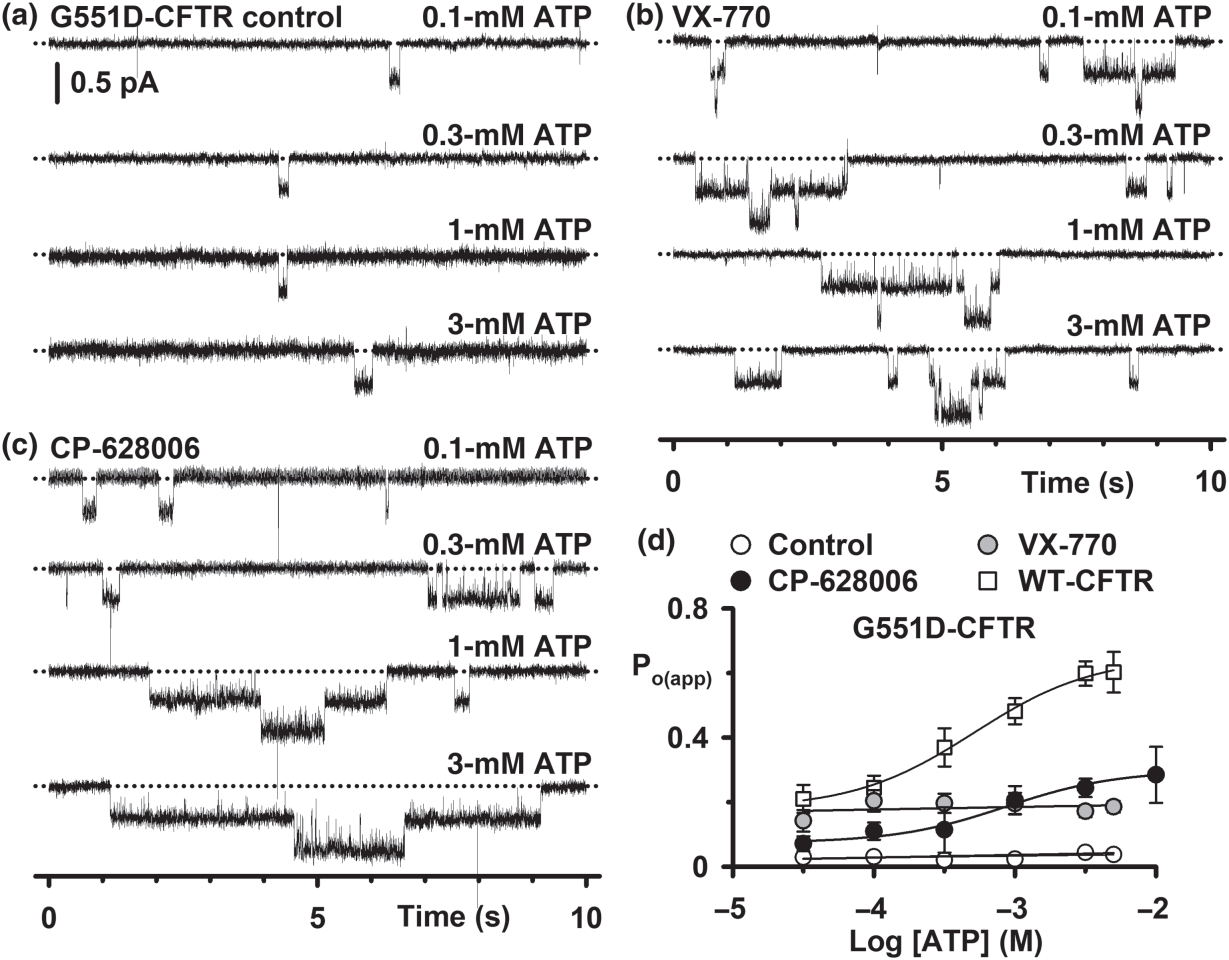
Potentiation of G551D‐CFTR by CP‐628006, but not ivacaftor, is ATP‐dependent. (a–c) Representative single‐channel recordings of G551D‐CFTR acquired using different membrane patches excised from HEK293 cells show the effects of the indicated intracellular ATP concentrations on potentiation by ivacaftor (VX‐770; 10 μM) and CP‐628006 (10 μM). PKA (75 nM) was continuously present in the intracellular solution. Dotted lines indicate the closed channel state and downward deflections correspond to channel openings. (d) Relationship between intracellular ATP concentration and P_o(app)_ for G551D‐CFTR in the absence and presence of either CP‐628006 (10 μM) or ivacaftor (10 μM). For comparison, the relationship between intracellular ATP‐concentration and P_o_ of wild‐type CFTR phosphorylated with PKA (75 nM) is shown. Data are means ± SEM (wild‐type, *n* = 5–7; G551D‐CFTR: control, *n* = 3; CP‐628006, *n* = 4–9; ivacaftor, *n* = 5–11). The continuous lines are the fit of first‐order regression (control and ivacaftor) and sigmoidal concentration‐response (CP‐628006) functions to mean data. In (d), the wild‐type CFTR data are the same as Figure 5. Other details as in Figure 2

**FIGURE 7:**
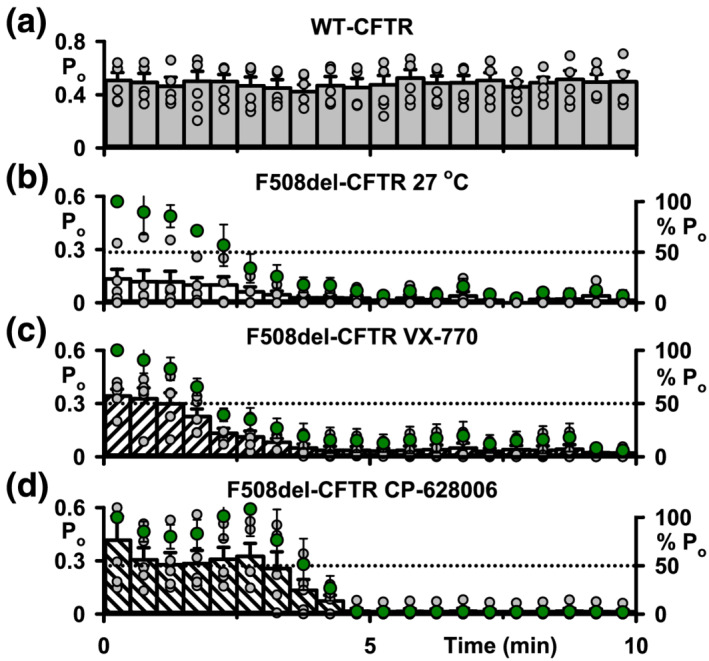
CP‐628006 delays, but does not prevent, loss of low temperature‐rescued F508del‐CFTR function in cell‐free membrane patches. (a–d) Time courses of P_o_ for wild‐type CFTR and low‐temperature‐rescued F508del‐CFTR Cl^−^ channels in excised inside‐out membrane patches commenced once channel activation was complete. ATP (1 mM) and PKA (75 nM) were continuous present in the intracellular solution. In (b)–(d), left and right ordinates show P_o_ (bars) and normalised P_o_ (green circles), respectively. Wild‐type CFTR Cl^−^ channels (a) were studied at 37°C, whereas F508del‐CFTR Cl^−^ channels (b) were activated at 27°C before temperature was increased to 37°C and P_o_ measured. In (c) and (d), following activation of F508del‐CFTR Cl^−^ channels at 27°C, they were potentiated with either ivacaftor (VX‐770; 1 μM) (c) or CP‐628006 (10 μM) (d) before temperature was increased to 37°C and P_o_ measured. Grey circles represent individual values and columns are means ± SEM (*n* = 5). Other details as in Figure 2

**FIGURE 8:**
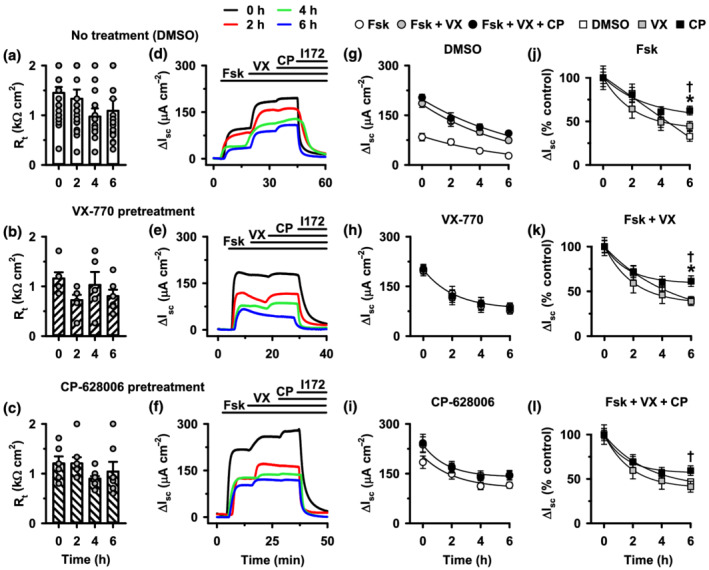
CP‐628006 slows the loss of F508del‐CFTR stability in polarised epithelia. (a–c) Transepithelial resistance (R_t_) and (d–f) representative Ussing chamber recordings of low temperature‐rescued F508del‐CFTR expressing FRT epithelia. Fifteen minutes prior to *t* = 0 h, FRT epithelia were treated with cycloheximide (50 μg·ml^−1^) and DMSO (0.1% v·v^−1^) (a, d), ivacaftor (VX‐770; 1 μM) (b, e), or CP‐628006 (10 μM) (c, f) added to both the apical and basolateral solutions. At the indicated times, FRT epithelia were mounted in Ussing chambers and CFTR Cl^−^ currents activated with forskolin (Fsk; 10 μM), potentiated with ivacaftor (VX‐770 [VX]; 1 μM) and CP‐628006 (CP; 10 μM) and inhibited with CFTR_inh_‐172 (I172; 10 μM); continuous lines indicate the presence of different compounds in the apical solution; cycloheximide (50 μg·ml^−1^) was present in the apical and basolateral solutions during I_sc_ recordings. Data are normalised to baseline current so that ΔI_sc_ represents the change in transepithelial current after CFTR activation by forskolin. (g–i) Magnitude of forskolin‐stimulated (Fsk), ivacaftor‐potentiated (Fsk + VX) and total current (ivacaftor‐ and CP‐628006‐potentiated; Fsk + VX + CP) of low temperature‐rescued F508del‐CFTR expressing FRT epithelia at different times after treatment with cycloheximide and DMSO or small molecules. (j–l) Normalised magnitude of forskolin‐stimulated (j), ivacaftor‐potentiated (k) and ivacaftor and CP‐628006‐potentiated (l) for FRT epithelia treated with cycloheximide and DMSO, ivacaftor, or CP‐628006. In (a)–(c) and (g)–(l), data are means ± SEM (DMSO, *n* = 8–10; ivacaftor, *n* = 5–6; CP‐628006, *n* = 6–8); *P < 0.05 CP‐628006 versus DMSO; ^†^ *P* < 0.05 CP‐628006 versus ivacaftor; one‐way ANOVA with Fisher's least significant difference post hoc test. Error bars are smaller than symbol size except where shown. In (a)–(c), symbols represent individual values, and in (g)–(l), continuous lines are the fit of single exponential functions to mean data. (j) Normality test (Shapiro–Wilk), *P* = 0.395 (passed); equal variance test (Brown‐Forsythe), *P* = 0.727 (passed); (k) normality test (Shapiro–Wilk), *P* = 0.691 (passed); equal variance test (Brown‐Forsythe), *P* = 0.618 (passed); (l) normality test (Shapiro–Wilk), *P* = 0.504 (passed); equal variance test (Brown‐Forsythe), *P* = 0.495 (passed))

The data and statistical analyses used in this study comply with the recommendations on experimental design and data analysis in pharmacology (Curtis et al., 2018), with the exception that some group sizes were unequal due to technical difficulties with the acquisition of single‐channel data. Data recording and analyses were randomised, but not blinded and to avoid pseudo‐replication, all experiments were repeated at different times. Results are expressed as means ± SEM of *n* observations. In halide flux studies (Figure 1), *n* represents number of replicates times days tested, with two replicates tested per day; in Ussing chamber studies, *n* represents either the number of days tested with three to four epithelia tested per day (Figure 1) or the number of epithelia (Figure 8); in automated electrophysiology (Figure S2), *n* represents the number of cells and in single‐channel studies (Figures 2, 3, 4, 5, 6, 7 and S1) *n* represents the number of membrane patches from different cells.

Data subject to statistical analysis had *n* values ≥ 5 per group and were tested for normal distribution using a Shapiro–Wilk normality test; differences were considered statistically significant when *P* < 0.05. To test for differences between two groups of data acquired within the same experiment, we used Student's paired *t*‐test. To test for differences between multiple groups of data, we used a one‐way analysis of variance (ANOVA), and where *F* < 0.05, post hoc tests were performed (Figure 3a,b, Dunnett's; Figures 4e and S2D, Holm‐Sidak; Figure 8j–l, Fisher's least significant difference). For the data in Figures 3c and 4d,f that failed either normality or equal variance tests, a Kruskal‐Wallis one‐way analysis of variance on ranks was performed. Tests were performed using Excel (version 13.0; Microsoft Corp., Redmond, WA, USA) (RRID:SCR_016137) and SigmaPlot™ (version 13.0; Systat software Inc., San Jose, CA, USA) (RRID:SCR_010285).

### 2.7 Nomenclature of targets and ligands

Key protein targets and ligands in this article are hyperlinked to corresponding entries in the IUPHAR/BPS Guide to PHARMACOLOGY http://www.guidetopharmacology.org and are permanently archived in the Concise Guide to PHARMACOLOGY 2021/22 (Alexander et al., 2021).

## 3 RESULTS

### 3.1 Identification and initial characterisation of the CFTR potentiator CP‐628006

Exploratory data. As part of a cystic fibrosis drug discovery programme, a high‐throughput screen of approximately 150,000 small molecules was performed to identify new CFTR potentiators. This screen used FRT cells co‐expressing low temperature‐rescued F508del‐CFTR and halide‐sensitive yellow fluorescent protein (YFP) in an automated microtitre plate‐based halide flux assay. After confirming and triaging hits, CP‐628006 was identified as a new CFTR potentiator with a chemical structure distinct from known CFTR potentiators, such as ivacaftor (Figure 1a). In this cellular system, CP‐628006 potentiated low temperature‐rescued F508del‐CFTR with an EC_50_ of 0.4 μM and a maximal response approximately half that of ivacaftor (Figure 1b).

To evaluate the potency and efficacy of CP‐628006, we tested its effects on heterologously expressed and native CFTR with the Ussing chamber technique. To specifically study CFTR function in the apical membrane of FRT epithelia heterologously expressing F508del‐ and G551D‐CFTR, we measured apical Cl^−^ currents (I_Cl_^apical^) after permeabilising the basolateral membrane with amphotericin B, whereas to study native CFTR, we measured CFTR‐mediated short‐circuit current (I_sc_) in epithelia of human bronchial epithelial cells from cystic fibrosis patients (genotypes: F508del/F508del and F508del/G551D). To minimise current deactivation caused by the thermal instability of F508del‐CFTR (Aleksandrov et al., 2010; Liu et al., 2012; Wang et al., 2011), we acquired F508del‐CFTR data at 27°C. Figure 1c,d shows concentration–response relationships for lumacaftor‐rescued F508del‐CFTR and G551D‐CFTR in FRT epithelia potentiated by CP‐628006 and ivacaftor, while Figure 1e,f shows equivalent data for epithelia of human bronchial epithelial cells from cystic fibrosis patients (genotypes: F508del/F508del and F508del/G551D). Table 1 compares the potency and efficacy of CP‐628006 and ivacaftor calculated from these data. As expected for the comparison of a screening hit with a marketed drug, the potency and efficacy of CP‐628006 were lower than those of ivacaftor (Table 1). The data also suggest that CP‐628006 potentiates F508del‐CFTR with greater potency and efficacy than it potentiates G551D‐CFTR (Table 1). Because CP‐628006 efficaciously potentiated mutant CFTR in human bronchial epithelial cells and because its chemical structure was distinct from previously reported CFTR potentiators, we selected it for detailed investigation following these exploratory studies.

### 3.2 CP‐628006 potentiates the single‐channel gating of wild‐type, F508del‐, and G551D‐CFTR

To learn how CP‐628006 potentiates CFTR, we studied CFTR Cl^−^ channels in excised inside‐out membrane patches (Figures 2 and S1). Figures 2 and S1 demonstrate that CP‐628006 (10 nM–10 μM) was without effect on current flow through open channels, but enhanced markedly channel gating. For wild‐type CFTR, the gating pattern is characterised by bursts of channel openings interrupted by brief flickery closures, separated by longer closures between bursts (Figure S1A,B). Increasing the concentration of either CP‐628006 or ivacaftor in the intracellular solution enhanced the frequency and duration of channel openings and hence, P_o_ (Figures 2c and S1A,B). Using ATP (0.3 mM), CP‐628006 and ivacaftor potentiated wild‐type CFTR with similar efficacy, but ivacaftor had a 100‐fold greater potency (CP‐628006: EC_50_, 24 nM; maximum P_o_ [P_o max_], 0.77; *r* ^2^, 0.99; ivacaftor: EC_50_, 0.21 nM; P_o max_, 0.71; *r* ^2^, 0.99) (Figure 2c).

Consistent with previous results (e.g. Bompadre et al., 2007; Cai et al., 2006; Meng et al., 2017; Miki et al., 2010), low temperature‐rescued F508del‐ and G551D‐CFTR exhibited severe gating defects characterised by infrequent channel openings separated by very prolonged channel closures, but were without effect on current flow through open channels, prior to channel deactivation (Figures 2a,b and S1C,D). Like their effects on wild‐type CFTR, raising the concentration of CP‐628006 and ivacaftor increased markedly the frequency and duration of F508del‐ and G551D‐CFTR channel openings (Figures 2a,b and S1C,D). Of note, the P_o_ of F508del‐CFTR in the presence of the compounds achieved values equal to those of wild‐type CFTR in the absence of CFTR potentiators, while the P_o(app)_ values of G551D‐CFTR were noticeably increased, but not increased to wild‐type levels (Figure 2c–e). Using ATP (1 mM), CP‐628006 potentiated F508del‐ and G551D‐CFTR with slightly greater efficacy than ivacaftor, but ivacaftor had greater potency than CP‐628006, albeit this difference was greatly reduced with G551D‐CFTR (F508del‐CFTR CP‐628006: EC_50_, 400 nM; P_o max_, 0.62; *r* ^2^, 1.0; F508del‐CFTR ivacaftor: EC_50_, 10 nM; P_o max_, 0.51; *r* ^2^, 1.0; G551D‐CFTR CP‐628006: EC_50_, 380 nM; P_o(app) max_, 0.38; *r* ^2^, 0.97; G551D‐CFTR ivacaftor: EC_50_, 140 nM; P_o(app) max_, 0.31; *r* ^2^, 0.99) (Figure 2d,e and Table 1).

We acquired a small number of excised membrane patches with only one active low temperature‐rescued F508del‐CFTR Cl^−^ channel. Using these data, we performed an analysis of bursts to determine how maximally effective concentrations of CP‐628006 and ivacaftor potentiate F508del‐CFTR. Consistent with Figure 2a, Figure 3 demonstrates that the principal effects of both CP‐628006 (10 μM) and ivacaftor (1 μM) were to reduce the interburst interval (IBI) 15‐fold to approach the IBI value of wild‐type CFTR under similar conditions in the absence of potentiators (Wang et al., 2018). Both compounds also prolonged mean burst duration (MBD), albeit the effect of ivacaftor (1 μM) was modest compared to that of CP‐628006 (10 μM) (Figure 3). Thus, by increasing the frequency and duration of channel openings, CP‐628006 is an efficacious CFTR potentiator.

### 3.3 Combinations of CP‐628006 and ivacaftor enhance the potentiation of G551D‐CFTR, but not F508del‐CFTR

Previous studies have investigated the potential for combinations of CFTR potentiators to restore greater function to CFTR variants than individual compounds (termed co‐potentiation) (Phuan et al., 2018; 2019). To investigate whether CP‐628006 and ivacaftor co‐potentiate F508del‐ and G551D‐CFTR, we used compound concentrations that maximally potentiate single‐channel activity (Figure 2d,e). Because of the slow dissociation of ivacaftor from CFTR and its aqueous solubility limitations, which prevents reversal of its action (Csanády & Töröcsik, 2019), we added CP‐628006 prior to ivacaftor.

Figure 4a–c demonstrates the acute effects of CP‐628006 and ivacaftor by themselves and together on plasma membrane‐rescued F508del‐ and G551D‐CFTR Cl^−^ channels. The P_o_ of low temperature‐rescued F508del‐CFTR was enhanced 13 ± 5‐fold by CP‐628006 (10 μM), 9 ± 5‐fold by ivacaftor (1 μM), but only 14 ± 8‐fold by CP‐628006 (10 μM) and ivacaftor (1 μM) (Figure 4d); comparable results were obtained using lumacaftor, the first CFTR corrector approved for clinical use (Van Goor et al., 2011; Wainwright et al., 2015), to rescue F508del‐CFTR (Figure 4e). Thus, consistent with previous results (Kopeikin et al., 2014; Meng et al., 2017; Wang et al., 2018), the single‐channel behaviour of F508del‐CFTR delivered to the plasma membrane by either low temperature or lumacaftor was equivalent. Like their effects on individual F508del‐CFTR Cl^−^ channels (Figure 4d,e), CP‐628006 (1 μM) and ivacaftor (0.02 μM) together did not enhance the CFTR‐mediated I_sc_ generated by basal F508del‐CFTR expression in epithelia of human bronchial epithelial cells (genotype: F508del/F508del) greater than that achieved by ivacaftor (0.02 μM) alone in exploratory studies (*n* = 2; data not shown).

Figure 4f shows that the P_o(app)_ of G551D‐CFTR was enhanced 71 ± 30‐fold by CP‐628006 (10 μM), 44 ± 12‐fold by ivacaftor (10 μM), and 101 ± 33‐fold by CP‐628006 (10 μM) and ivacaftor (10 μM). Using automated planar patch‐clamp recording, application of both compounds together achieved noticeably larger G551D‐CFTR Cl^−^ currents than either CFTR potentiator applied alone at its maximally‐efficacious concentration (Figure S2). Thus, potentiation of G551D‐CFTR, but not F508del‐CFTR, was enhanced by combinations of CP‐628006 and ivacaftor tested at their maximally‐effective concentrations.

### 3.4 CP‐628006 restores ATP‐dependent channel gating to G551D‐CFTR

Both the F508del and G551D variants attenuate the ATP‐dependence of CFTR channel gating with the impact of G551D being especially severe (Bompadre et al., 2007; Lin et al., 2014; Schultz et al., 1999). Because ivacaftor potentiates both ATP‐dependent and ATP‐independent channel gating (Eckford et al., 2012; Jih & Hwang, 2013), we were particularly interested to learn the effects of CP‐628006 on the ATP‐dependence of F508del‐ and G551D‐CFTR channel gating. For these experiments, we used compound concentrations that robustly potentiated F508del‐ and G551D‐CFTR at ATP (1 mM) (Figure 2d,e) and acquired control data with different membrane patches to those used to study CP‐628006 and ivacaftor.

Figure 5 shows representative recordings of low temperature‐rescued F508del‐CFTR Cl^−^ channels in the absence and presence of either CP‐628006 (300 nM) or ivacaftor (7 nM) using ATP (0.1–3 mM) in the intracellular solution. As the ATP concentration was raised, F508del‐CFTR channel activity increased, but it was noticeably greater in the presence of either CP‐628006 (300 nM) or ivacaftor (7 nM) (wild‐type CFTR: EC_50_, 0.37 mM; P_o max_, 0.65; *r* ^2^, 1.0; F508del‐CFTR control: EC_50_, 0.55 mM; P_o max_, 0.08; *r* ^2^, 0.98; F508del‐CFTR CP‐628006: EC_50_, 0.29 mM; P_o max_, 0.33; *r* ^2^, 1.0; F508del‐CFTR ivacaftor: EC_50_, 0.11 mM; P_o max_, 0.26; *r* ^2^, 1.0) (Figure 5). These data suggest that CP‐628006 and ivacaftor have closely comparable effects, both increasing the affinity and efficacy of F508del‐CFTR channel gating by ATP, albeit at the concentrations tested, they did not restore the ATP‐dependence of wild‐type CFTR.

Figure 6 shows representative recordings of G551D‐CFTR Cl^−^ channels in the absence and presence of either CP‐628006 (10 μM) or ivacaftor (10 μM) using ATP (0.1–3 mM) in the intracellular solution. Consistent with previous results (Bompadre et al., 2007; Eckford et al., 2012; Jih & Hwang, 2013), in both the absence and presence of ivacaftor, the activity of G551D‐CFTR was unaffected by altering the ATP concentration (Figure 6). G551D‐CFTR channel activity was extremely low under control conditions, but was potentiated robustly by ivacaftor at all ATP concentrations tested (Figure 6). By contrast, as the ATP concentration was raised in the presence of CP‐628006 (10 μM), there was a noticeable increase in G551D‐CFTR activity (G551D‐CFTR CP‐628006: EC_50_, 0.86 mM; P_o(app) max_, 0.30; *r* ^2^, 0.98) (Figure 6). Thus, CP‐628006 and ivacaftor have markedly different effects on G551D‐CFTR channel gating with CP‐628006, but not ivacaftor, conferring the mutant with ATP‐dependence.

### 3.5 CP‐628006, delays, but does not prevent, the deactivation of F508del‐CFTR

Some CFTR potentiators, including ivacaftor, accentuate the plasma membrane instability of F508del‐CFTR, whereas others, including ΔF508_act_‐02, do not (Cholon et al., 2014; Phuan et al., 2015; Veit et al., 2014; Yang et al., 2003). To investigate the effects of CP‐628006 on the plasma membrane instability of F508del‐CFTR, we monitored the duration of channel activity in excised inside‐out membrane patches at 37°C in the continuous presence of PKA (75 nM) and ATP (1 mM) by measuring P_o_ once channels were fully activated by PKA‐dependent phosphorylation.

Figure 7a demonstrates that wild‐type CFTR exhibited robust, sustained channel activity characterised by P_o_ values of ~0.5. By contrast, the greatly reduced channel activity of low temperature‐rescued F508del‐CFTR was unstable, declining from P_o_ values of ~0.15 to zero within 8 min (Figure 7b). Consistent with previous results (e.g., Meng et al., 2017), acute potentiation of low temperature‐rescued F508del‐CFTR with ivacaftor (1 μM) enhanced greatly initial P_o_ values, but they then declined to zero noticeably more quickly (Figure 7c). Figure 7d demonstrates that potentiation of low temperature‐rescued F508del‐CFTR by CP‐628006 (10 μM) was more sustained than with ivacaftor (1 μM), delaying channel deactivation. Analysis of the time required to reach the normalised P_o_ value of 50% indicates that ivacaftor accelerated F508del‐CFTR deactivation in excised inside‐out membrane patches by ~30 s (approximately 22% decrease relative to low temperature‐rescued), whereas CP‐628006 delayed it by ~90 s (approximately 67% increase relative to low temperature‐rescued) (Figure 7).

To investigate further the action of CP‐628006 on the plasma membrane instability of F508del‐CFTR, we studied CFTR‐mediated I_sc_ in low temperature rescued‐F508del‐CFTR‐expressing FRT epithelia treated for 6 h with test compounds or the vehicle DMSO. To specifically investigate the CFTR population in the apical membrane, we also treated FRT epithelia with the protein synthesis inhibitor cycloheximide (50 μg·ml^−1^) at the time of compound addition. These treatments were without effect on R_t_ (Figure 8a–c), suggesting that epithelial integrity was unaffected over the period of study.

**TABLE 1:** Potency and efficacy of CP‐628006 and ivacaftor in epithelia, cells and channels

Experimental preparation	CP‐628006		Ivacaftor	
	EC_50_ (μM) (95% CI)	Maximal effect (%) (95% CI)	EC_50_ (μM) (95% CI)	Maximal effect (%) (95% CI)
F508del‐CFTR FRT epithelia^a^ ^,^ ^b^	ND^c^	ND^c^	0.002 (0.002 to 0.002)	103 (101.9 to 103.3)
G551D‐CFTR FRT epithelia^b^	1.68^d^	39^d^	0.003 (0.002 to 0.005)	97 (89.5 to 107.3)
F508del/F508del epithelia of hBE cells^b^ ^,^ ^e^	0.062 (0.054 to 0.073)	56 (54.4 to 58.4)	0.002 (0.001 to 0.003)	102 (89.7 to 117.3)
F508del/G551D epithelia of hBE cells^f^	0.14^d^	18^d^	0.005 (0.003 to 0.016)	103 (87.5 to 130.2)
G551D‐CFTR HEK cells^g^	5.13 (3.00 to 9.16)	72 (52.9 to 99.2)	0.82 (0.27 to 2.41)	70 (50.3 to 108.8)
F508del‐CFTR Cl^−^ channels^h^	0.50 (0.18 to 1.43)	143.8 (110.9 to 181.8)	0.008 (0.003 to 0.026)	102.6 (85.6 to 121.3)
G551D‐CFTR Cl^−^ channels^h^	0.17 (0.03 to 0.68)	144.8 (119.1 to 174.5)	0.036 (0.006 to 0.117)	96.7 (67.8 to 134.8)

*Note*: Calculated EC_50_ and maximal effect values from concentration–response relationships for CP‐628006 and ivacaftor potentiation of F508del‐ and G551D‐CFTR in hBE cells expressing native CFTR and FRT and HEK cells heterologously expressing CFTR determined by least squares fitting as described in the legends of Figures 1, 2, and S2. In FRT epithelia, I_Cl_^apical^ was measured (Figure 1c,d), whereas in epithelia from hBE cells, I_sc_ was recorded (Figure 1e,f). In HEK cells, whole‐cell currents were studied by automated electrophysiology (Figure S2). F508del‐ and G551D‐CFTR Cl^−^ channels were studied in excised inside‐out membrane patches (Figure 2c–e).

Abbreviation: CI, confidence interval, hBE cells, human bronchial epithelial cells.

^a^ F508del‐CFTR expression was rescued with lumacaftor (5 μM) at 37°C for 18–24 h.

^b^ I_Cl_^apical^ from FRT epithelia and I_sc_ from hBE cell‐derived epithelia was studied at 27°C to minimise the thermal instability of mutant CFTR.

^c^ CP‐628006 tested at concentrations ≤1 μM. Plateau effect not reached; EC_50_ > 0.5 μM.

^d^ Confidence intervals could not be determined.

^e^ Basal F508del‐CFTR expression was used for studies of hBE cell‐derived epithelia; I_sc_ data were acquired at 27°C to minimise the thermal instability of mutant CFTR.

^f^ I_sc_ from F508del/G551D hBE epithelia was studied at 35°C.

^g^ CFTR‐mediated whole‐cell currents were studied at 23°C.

^h^ F508del‐ and G551D‐CFTR Cl^−^ channels were studied at 23°C.

For DMSO‐treated F508del‐CFTR, at *t* = 0 h, forskolin (10 μM) stimulated modest CFTR‐mediated I_sc_, which was potentiated two‐fold by acute addition of ivacaftor (1 μM) after which CP‐628006 (10 μM) had little additional effect, but all current was inhibited by CFTR_inh_‐172 (10 μM) (Figure 8d). Consistent with previous results (Meng et al., 2017), for ivacaftor‐treated F508del‐CFTR, at *t* = 0 h, forskolin (10 μM) stimulated large CFTR‐mediated I_sc_, which was not further potentiated by acute addition of either ivacaftor (1 μM) or CP‐628006 (10 μM) (Figure 8e). Similarly, for CP‐628006‐treated F508del‐CFTR, at *t* = 0 h, forskolin (10 μM) stimulated large CFTR‐mediated I_sc_, but this was further potentiated by acute addition of ivacaftor (1 μM) after which CP‐628006 (10 μM) had little additional effect (Figure 8f). Figure 8g–i shows the magnitude of forskolin‐stimulated, ivacaftor‐potentiated or ivacaftor and CP‐628006‐potentiated CFTR‐mediated I_sc_ at different times after FRT epithelia were treated with CFTR potentiators and cycloheximide. For F508del‐CFTR FRT epithelia treated with CP‐628006 or ivacaftor, the magnitude of forskolin‐stimulated CFTR‐mediated I_sc_ was enhanced greatly at *t* = 0 h compared to those treated with DMSO, but over the 6 h time course I_sc_ magnitude declined under all conditions tested (Figure 8g–i). Nevertheless, the decline of CFTR‐mediated I_sc_ was slower with CP‐628006 (Figure 8j–l). Taken together, the data demonstrate that CFTR potentiation by CP‐628006 shows important differences from the action of ivacaftor.

## 4 DISCUSSION

This study investigated the action of CP‐628006, a CFTR potentiator with a chemical structure distinct from previously reported potentiators. CP‐628006 efficaciously potentiated the CFTR‐mediated Cl^−^ currents of plasma membrane‐rescued F508del‐ and G551D‐CFTR. Its divergent effects compared to those of ivacaftor suggest a different mechanism of action.

Many CFTR potentiators have been identified by hypothesis‐driven studies (e.g. phloxine B, Cai & Sheppard, 2002; capsaicin, Ai et al., 2004) and high‐throughput screening (e.g., ΔF508_act_‐02, Yang et al., 2003; ivacaftor, Van Goor et al., 2009). However, few CFTR potentiators have been tested in the clinic and until recently only ivacaftor was approved for cystic fibrosis patient use (Becq et al., 2011; Ramsey et al., 2011; Ramsey & Welsh, 2017). The CFTR corrector elexacaftor, part of the triple combination therapy elexacaftor‐tezacaftor‐ivacaftor (Trikafta) (Heijerman et al., 2019; Middleton et al., 2019), is now recognised to display both corrector and potentiator activity (Laselva et al., 2021; Veit et al., 2021). Like CP‐628006 potentiation by elexacaftor appears distinct to that of ivacaftor, as its action is additive to other potentiators (Veit et al., 2021). Among other CFTR potentiators tested in the clinic, CTP‐656 (VX‐561, deuterated ivacaftor) (Ramsey & Welsh, 2017) is expected to act like ivacaftor. Similar to ivacaftor, the mechanism of action of GLPG1837 is independent of the control of channel gating by ATP binding and hydrolysis at the nucleotide‐binding domains (NBDs) (Yeh et al., 2017). By contrast, genistein potentiates CFTR channel gating by binding at the NBD1:NBD2 interface, where it accelerates channel opening and delays channel closure (Ai et al., 2004; Moran et al., 2005). Analysis of chemical structures demonstrates that diverse chemical scaffolds function as CFTR potentiators (Becq et al., 2011). The chemical structure of CP‐628006 (rac‐(4*bR*,7*R*,8*aS*)‐4b‐benzyl‐7‐hydroxy‐*N*‐((2‐methylpyridin‐3‐yl)methyl)‐7‐(3,3,3‐trifluoropropyl)‐4*b*,5,6,7,8,8*a*,9,10‐octahydrophenanthrene‐2‐carboxamide) is different from other known potentiators, adding to the structural diversity of CFTR potentiators. This work suggests that it also adds to the mechanistic diversity of CFTR potentiators.

We examined the potentiation of F508del‐ and G551D‐CFTR by CP‐628006 and ivacaftor at the molecular, cellular, and tissue levels using single‐channel recording, automated planar patch recording, and the Ussing chamber technique. Consistent with their identities as a marketed drug and a screening hit, ivacaftor was more potent than CP‐628006 in all assays (Table 1). Although the efficacy of ivacaftor also exceeded that of CP‐628006 in polarised epithelia, the two compounds had similar efficacy in single cells, while CP‐628006 potentiated individual CFTR Cl^−^ channels with greater efficacy than ivacaftor (Table 1). The data also suggest that ivacaftor and CP‐628006 restored wild‐type levels of channel activity (as judged by P_o_ values) to F508del‐CFTR. It was not possible to make a similar conclusion for G551D‐CFTR because unobserved channels prevented accurate determination of P_o_ (for discussion, see Cai et al., 2011). Moreover, in single cells, co‐potentiation of G551D‐CFTR by CP‐628006 and ivacaftor was stronger than in cell‐free membrane patches. One possible explanation for these differences is that in polarised epithelia and single cells, CFTR function is influenced by the activity of ion channels, transporters, and interacting proteins that establish and/or modify the electrochemical gradients for transmembrane ion movements, whereas in excised inside‐out membrane patches, CFTR function is studied directly. A further possibility is differences in CFTR phosphorylation between intact cells and cell‐free membrane patches (Cui et al., 2019; Pyle et al., 2011). Finally, other factors arising from differences in experimental conditions might also account for the divergent results.

An important finding of this study is the distinct actions of CP‐628006 and ivacaftor on CFTR channel gating. Once CFTR is phosphorylated by PKA, ivacaftor enhances CFTR activity by potentiating both ATP‐dependent and ATP‐independent channel gating (Eckford et al., 2012; Jih & Hwang, 2013). Using the energetic coupling model of CFTR channel gating (Jih et al., 2012), Jih and Hwang (2013) demonstrated that ivacaftor promotes gating transitions that favour channel open states, providing an explanation for the drug's effects on ATP‐dependent and ATP‐independent channel gating. Consistent with these data, the ivacaftor‐binding site is located at the interface of the membrane‐spanning domains and the lipid bilayer and involves residues located in transmembrane segments 4 and 5 (M4 and M5) and the unstructured region of M8 (Liu et al., 2019; Yeh et al., 2019). Although we did not assess directly the effects of CP‐628006 on ATP‐independent channel gating, its restoration of ATP‐dependence to G551D‐CFTR, a CFTR variant almost unresponsive to ATP (Lin et al., 2014), suggests that CP‐628006 predominantly acts by enhancing ATP‐dependent channel gating. Because G551D obstructs conformational changes following ATP binding at the canonical ATP‐binding site (site 2) (Bompadre et al., 2007), the simplest interpretation of our data is that CP‐628006 interacts directly with site 2 to potentiate channel gating by ATP‐driven NBD dimerisation (Vergani et al., 2003; 2005). CP‐628006 might increase the frequency and duration of channel openings by providing binding energy to promote NBD1:NBD2 dimerisation and stabilise the interaction of ATP with site 2. Alternatively, it might bind at a remote site, such as the NBD1:NBD2 interface, another binding site for CFTR potentiators (Kalid et al., 2010; Moran et al., 2005) and enhance allosterically the interaction of ATP with site 2. Interestingly, Xu et al. (2014) showed that the revertant mutation 4RK (the simultaneous mutation of four arginine‐framed tripeptides [R29K, R516K, R555K, and R766K]) restored some ATP‐dependence to G551D‐CFTR. Thus, future studies of 4RK might provide insight into how CP‐628006 potentiates CFTR channel gating.

As exemplified by F508del, many pathogenic variants destabilise CFTR at the plasma membrane (Veit et al., 2016), reducing thermal stability (e.g., Aleksandrov et al., 2010) and accelerating endocytosis (e.g. Swiatecka‐Urban et al., 2005). Previous work suggests that these CFTR variants are susceptible to accelerated plasma membrane destabilisation by CFTR potentiators, including ivacaftor, which perturbs CFTR structure and disrupts the lipid bilayer (Avramescu et al., 2017; Chin et al., 2018; Cholon et al., 2014; Veit et al., 2014). They also suggest that the destabilising effects of CFTR potentiators is mutation‐specific (Avramescu et al., 2017), arguing that new CFTR potentiators will need to be screened against individual CFTR variants to identify compounds without adverse effects on stability. Encouragingly, the present results show that CP‐628006 delays the deactivation of F508del‐CFTR in excised membrane patches and polarised epithelia. Moreover, we did not observe any evidence of CFTR inhibition by high concentrations of CP‐628006, unlike some CFTR potentiators, which impede channel gating and/or occlude the channel pore at elevated concentrations (e.g., genistein; Lansdell et al., 2000). Thus, the example of CP‐628006 demonstrates that some CFTR potentiators can improve the plasma membrane stability of F508del‐CFTR and that their enhancement of channel gating is widely separated from any inhibitory effects.

The present results and previous work (e.g. Lin et al., 2016; Yu et al., 2011) highlight the potential of co‐potentiation to maximise the restoration of function to CFTR variants (Phuan et al., 2018). To explain the mechanism of co‐potentiation, Phuan et al. (2019) identified two classes of CFTR potentiators: class I potentiators (e.g., ivacaftor and GLPG1837), which share a common binding site at the membrane‐spanning domain‐lipid interface (Liu et al., 2019; Yeh et al., 2019), and class II potentiators (e.g. ASP‐11 and apigenin), which might bind at the NBD1:NBD2 dimer interface in a position distinct from the ATP‐binding sites (Bose et al., 2020; Moran et al., 2005). Co‐potentiation occurred when a class I potentiator was used with a class II potentiator, but not when two potentiators from the same class were employed (Phuan et al., 2019). Co‐potentiation by elexacaftor with both ivacaftor and apigenin (Veit et al., 2021) suggests that it might represent an additional class of potentiators. Intriguingly, some CFTR variants are receptive to co‐potentiation (e.g. G551D and S549N), whereas other CFTR variants are insensitive (e.g. A561E and M1101K) (Phuan et al., 2019; Veit et al., 2020). Consistent with these data, we found that ivacaftor and CP‐628006 co‐potentiated G551D‐CFTR, but not F508del‐CFTR.

In conclusion, CP‐628006 is an efficacious CFTR potentiator with a chemical structure unlike any previously identified CFTR potentiator, which robustly restores channel gating to F508del‐ and G551D‐CFTR in cells heterologously‐expressing CFTR and in patient‐derived primary cultures of human bronchial epithelial cells expressing native CFTR. Its distinct effects compared to ivacaftor suggest a different mechanism of action, which modifies the regulation of channel gating by ATP‐driven NBD dimerisation. The finding that combinations of CP‐628006 and ivacaftor potentiate G551D‐CFTR greater than either compound alone supports the conclusion of different modes of action. This additional example of co‐potentiation supports the concept that combinations of CFTR potentiators might have additional therapeutic benefit in cystic fibrosis.

## AUTHOR CONTRIBUTIONS

J.L., A.P.B., E.B.S., D.N.S, L.C. and M.J.P. were responsible for the conception and design of the experiments; J.L., A.P.B., Y.W. and W.J. performed the research; J.L., A.P.B., Y.W., W.J., K.J.S., E.B.S., D.N.S., L.C. and M.J.P. were responsible for the analysis and interpretation of data; J.L., A.P.B., K.J.S., D.N.S. and M.J.P. were responsible for drafting the article or revising it critically for important intellectual content. All authors approved the final version of the manuscript.

## CONFLICT OF INTEREST

A.P.B. and M.J.P. are employees of Pfizer Inc., L.C. and E.B.S. former employees, and J.L. was a Pfizer postdoctoral scientist. The contribution of L.C. was limited to his period of employment by Pfizer Inc. All other authors declare that they have no conflicts of interest with the contents of this article.

## DECLARATION OF TRANSPARENCY AND SCIENTIFIC RIGOUR

This Declaration acknowledges that this paper adheres to the principles for transparent reporting and scientific rigour of preclinical research as stated in the *BJP* guidelines for Design and Analysis and as recommended by funding agencies, publishers and other organisations engaged with supporting research.

## Supporting information


**Figure S1:** **Potentiation of wild‐type and G551D‐CFTR Cl^‐^ channels by CP‐628006** (A –D) Representative single‐channel recordings of wild‐type and G551D‐CFTR in excised inside‐out membrane patches from HEK293 cells show the effects of addition of the indicated concentrations of CP‐628006 and ivacaftor (VX‐770) to the intracellular solution. ATP (0.3 mM (wild‐type CFTR) or 1 mM (G551D‐CFTR)) and PKA (75 nM) were continuously present in the intracellular solution. Dotted lines indicate the closed channel state and downward deflections correspond to channel openings. Membrane patches were voltage‐clamped at −50 mV, a large Cl^‐^ concentration gradient imposed across the membrane ([Cl^‐^]_int_, 147 mM; [Cl^‐^]_ext_, 10 mM) and temperature was ~23 °C. For summary single‐channel concentration‐response relationships for wild‐type and G551D‐CFTR potentiated by CP‐628006 and ivacaftor, see Figure 2. Note that in Figure 2e, the ivacaftor (1 μM) data point is obscured behind that of CP‐628006 (1 μM).

**Figure S2:** **Potentiation of G551D‐CFTR‐mediated whole‐cell Cl^‐^ currents by CP‐628006, ivacaftor and combinations of CP‐628006 and ivacaftor** (A – C) Time‐courses of G551D‐CFTR‐mediated whole‐cell Cl^‐^ currents potentiated by maximally effective concentrations of CP‐628006 and ivacaftor either alone or together. The continuous lines indicate the presence of forskolin (10 μM), IBMX (100 μM), DMSO (1% vꞏv^−1^), CP‐628006 (CP; 10 μM), ivacaftor (VX; 10 μM) and CFTR_inh_‐172 (I172; 10 μM) in the extracellular solution. Membrane voltage was clamped at −60 mV and stepped to +60 mV to measure G551D‐CFTR‐mediated whole‐cell Cl^‐^ currents; temperature was ~23 °C. (D) Magnitude of G551D‐CFTR‐mediated whole‐cell Cl^‐^ current potentiated by CP‐628006 (10 μM) and ivacaftor (10 μM) either alone or together. Symbols represent individual values and columns are means ± SEM (CP‐628006, n = 6; ivacaftor, n = 14; CP‐628006 + ivacaftor, n = 14); *, *P* <  0.05 vs. ivacaftor; †, *P* <  0.05 vs. CP‐628006; one‐way ANOVA with Holm‐Sidak post‐hoc test. (E) Concentration‐response relationships for the potentiation of G551D‐CFTR‐mediated whole‐cell Cl^‐^ currents by CP‐ 628006 and ivacaftor (VX‐770). Data are means ± SEM (CP‐628006, n = 6–16; ivacaftor, n = 12–20). The continuous lines are the fit of sigmoidal concentration‐response functions to mean data. To determine EC_50_ and maximal effect values (Table 1), potentiation by CP‐628006 is expressed relative to that of ivacaftor with the ivacaftor concentration achieving maximal effect (10 μM) designated 100%. (Figure S2D: Normality Test (Shapiro–Wilk), *P* =  0.124 (passed); Equal Variance Test (Brown‐Forsythe), *P* =  0.092 (passed)).

## Data Availability

Data are available at the University of Bristol data repository, data.bris, at https://doi.org/10.5523/bris.39dh0wa8o0n482ww1p85wgrmqy.
